# Oligomeric Structural Transition of HspB1 from Chinese Hamster

**DOI:** 10.3390/ijms221910797

**Published:** 2021-10-06

**Authors:** Nina Kurokawa, Rio Midorikawa, Manami Nakamura, Keiichi Noguchi, Ken Morishima, Rintaro Inoue, Masaaki Sugiyama, Masafumi Yohda

**Affiliations:** 1 Department of Biotechnology and Life Science, Tokyo University of Agriculture and Technology, Tokyo 184-8588, Japan; nina.kurokawa@yohda.net (N.K.); rio.midorikawa@yohda.net (R.M.); manami.nakamura@yohda.net (M.N.); 2 Instrumentation Analysis Center, Tokyo University of Agriculture and Technology, Tokyo 184-8588, Japan; knoguchi@cc.tuat.ac.jp; 3 Institute for Integrated Radiation and Nuclear Science, Kyoto University, Osaka 590-0494, Japan; morishima@rri.kyoto-u.ac.jp (K.M.); inoue.rintaro.5w@kyoto-u.ac.jp (R.I.); sugiyama.masaaki.5n@kyoto-u.ac.jp (M.S.)

**Keywords:** chaperone, small heat shock protein, folding, Chinese hamster, analytical ultracentrifugation

## Abstract

HspB1 is a mammalian sHsp that is ubiquitously expressed in almost all tissues and involved in regulating many vital functions. Although the recent crystal structure of human HspB1 showed that 24 monomers form the oligomeric complex of human HspB1 in a spherical configuration, the molecular architecture of HspB1 is still controversial. In this study, we examined the oligomeric structural change of CgHspB1 by sedimentation velocity analytical ultracentrifugation. At the low temperature of 4 °C, CgHspB1 exists as an 18-mer, probably a trimeric complex of hexamers. It is relatively unstable and partially dissociates into small oligomers, hexamers, and dodecamers. At elevated temperatures, the 24-mer was more stable than the 18-mer. The 24-mer is also in dynamic equilibrium with the dissociated oligomers in the hexameric unit. The hexamer further dissociates to dimers. The disulfide bond between conserved cysteine residues seems to be partly responsible for the stabilization of hexamers. The N-terminal domain is involved in the assembly of dimers and the interaction between hexamers. It is plausible that CgHspB1 expresses a chaperone function in the 24-mer structure.

## 1. Introduction

Small heat shock proteins (sHsps) are members of molecular chaperones present in all kingdoms of life [1]. They protect cells from damage under various stress conditions. Mutations in human sHsps result in multiple diseases, myopathies, neuropathies, and cataracts [2]. In vitro, sHsps bind to partially folded or denatured proteins, preventing irreversible aggregation or promoting correct substrate folding. sHsps share a highly conserved α-crystallin domain (ACD) flanked by the N-terminal domain (NTD) and C-terminal extension (CTE) [1]. The CTE contains the characteristic three-residue IX(I/V) motif, which plays an essential role in oligomerization [3]. The NTD plays a driving role in oligomer formation and interactions with substrate proteins.

Most sHsps form large oligomeric structures composed of 12–36 subunits [4]. However, reports on the atomic structures of sHsp oligomers are limited. Archaeal sHsps exist as spherical 24-mer oligomers with a diameter of approximately 12 nm [5,6]. In contrast, the sHsp from wheat (wHsp16.9) forms a double-ring-shaped oligomer consisting of 12 subunits [7]. The sHsp from the fission yeast *Schizosaccharomyces pombe*, SpHsp16.0, forms a hexadecameric oligomer structure in which eight dimers of SpHsp16.0 form an elongated sphere with 422 symmetry [8]. Sip1 is one of 16 sHsps of *Caenorhabditis elegans*. The chaperone activity of Sip1 is regulated by pH [9]. Sip1 inhibited protein aggregation in a concentration-dependent manner under acidic conditions. The oligomeric structure of Sip1 changes with pH. Twenty-four-, 28-, and 32-mer Sip1 assemblies were observed by cryo-EM. At a low pH of 5.8, the oligomer changes to a relatively small oligomer structure. Among them, the crystal structure of the 32-mer was determined.

Ten sHsps have been identified in the human genome [10]. They have been categorized as Class I or Class II [2]. HspB1 (Hsp27), HspB5 (αB-crystallin), HspB6 (Hsp20), and HspB8 (Hsp22) are Class I sHsps that are distributed widely and found in various tissues. They are predominantly heat-inducible and play an essential role in cell survival under conditions of stress. Class II sHsps include HspB2, HspB3, HspB4 (αA-crystallin), HspB7, HspB9, and HspB10, which exhibit a tissue-restricted pattern of expression and are believed to play a significant role in development, differentiation, and specialized tissue-specific functions [11].

HspB1 is almost ubiquitously expressed in all human tissues [11,12] and regulates many vital functions. HspB1 is responsible for the regulation and stabilization of the cytoskeleton [13], possesses antiapoptotic activity [14], and protects the cell against oxidative stress [15]. Extracellular stresses induce phosphorylation of two or three serine residues [16]. HspB1 is known as Hsp27 in humans and Hsp25 in mice according to their apparent molecular mass.

Previously, we analyzed the oligomeric structure of HspB1 from CHO cells (CgHspB1) by size exclusion chromatography with multiangle light scattering (SEC-MALS) and small-angle X-ray scattering (SAXS). The results suggested that CgHspB1 has a 16-mer structure [17]. However, the molecular architecture of HspB1 is controversial. Analytical ultracentrifugation analysis showed that the mean molecular mass was 730 kDa [18]. Since the calculated molecular mass of human HspB1 is 22,782, it corresponds to a 32-mer. In contrast, HspB1/Hsp27 was reported to form a 24-mer by gel-filtration chromatography [19]. Lelj-Garolla et al. proposed that HspB1/Hsp27 exists in the equilibrium state of monomer/dimer, tetramer, 12-mer, and 16-mer based on sedimentation velocity analysis [20]. They also showed that the oligomerization of HspB1/Hsp27 increases with increasing temperature from 10 to 40 °C. The largest oligomers at 10 °C were 8–12-mer, whereas oligomers as large as 22–30-mer were observed at 40 °C [21]. Analytical size-exclusion chromatography (SEC) revealed that the wild-type Hsp27 eluted from the column as a broad peak, corresponding to an average molecular mass of ~590 kDa [22]. The molecular mass decreased by introducing phosphorylation mimic mutations. Mutations also increase chaperone activity. Therefore, it is reasonable to think that the dissociation of oligomers is correlated with molecular chaperone activity. Recently, the crystal structure of human HspB1 was reported [23]. Twenty-four monomers form the oligomeric complex of human HspB1 in a spherical configuration. Each monomer is constructed of a structurally conserved α-crystallin domain exhibiting a 6-stranded beta-sandwich, as previously described [24,25] with mobile N- and C-termini.

These results are not explained by the conventional model in which the oligomer of sHsp dissociates into dimers at elevated temperatures. It is plausible that HspB1 has various oligomeric structures that change with various conditions, temperature, concentration, or redox conditions. This study examined the oligomeric structural change of CgHspB1 by sedimentation velocity analytical ultracentrifugation (SV-AUC). Among the various methods to investigate the oligomeric structure of proteins, SV-AUC is most reliable for this purpose since this method can reveal the distribution of molecular weight or the association of oligomer precisely.

## 2. Results

### 2.1. Conformational Change of CgHspB1 Wild Type and S15D Mutant Analyzed by SV-AUC

We found that it was not easy to analyze the dissociated structures of CgHspB1 by SEC due to the interaction with the column. We could not observe dissociated conformers at elevated temperatures using the standard buffer. The problem was resolved by using a buffer containing 20% ethylene glycol [17]. At a high temperature of approximately 50 °C, CgHspB1 dissociated into small oligomers. The dissociation is reversible, and large oligomers reappear at lowered temperatures. We thought that the dissociated small oligomers were active structures. However, curiously, dissociated conformers were not observed at relatively high concentrations. We felt it challenging to elucidate the configuration change by SEC; therefore, we decided to analyze it by SV-AUC. Although it is unnecessary to consider the interaction with the column in SV-AUC, we used a buffer containing 20% ethylene glycol to avoid nonspecific interactions between CgHspB1 conformers.

**Figure 1:**
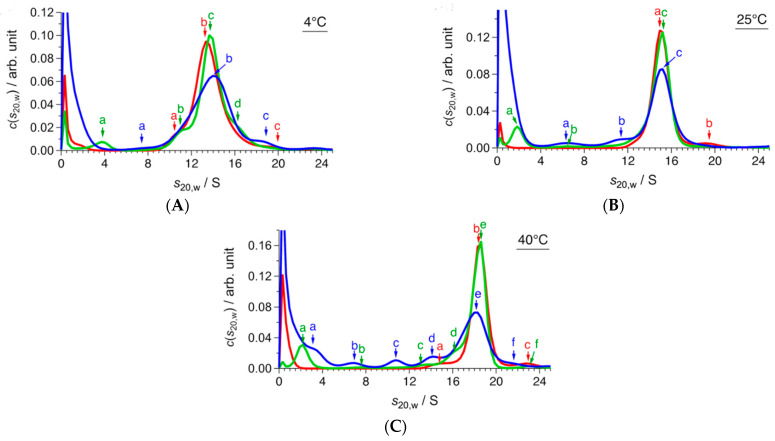
SV-AUC results of CgHspB1 WT at (**A**) 4 °C, (**B**) 25 °C, and (**C**) 40 °C. Red, green, and blue lines represent *c*(*s*_20,w_) at 1.5 mg/mL, 0.5 mg/mL, and 0.1 mg/mL, respectively. Colored arrows and letters (a, b, c, d, e, f) corresponding to the data with the same colors show the peak positions whose parameters are listed in Table 1.

Figure 1 shows the SV-AUC results of CgHspB1 wild-type (CgHspB1WT) depending on the temperature and concentration. In this study, the weight concentration distribution *c*(*s*_20,w_) and friction ratio *f*/*f*_0_ were derived from the sedimentation profiles by fitting analysis with the multicomponent Lamm equation. The molecular weight was calculated with Equation (1) (see Materials and Methods). Here, *f*/*f*_0_ was provided as the average value of all the components, meaning that *f*/*f*_0_ deviates from that of the main component in a polydisperse system. It should be noted that the deviation of *f*/*f*_0_ depends on the polydispersity of a sample. In the CgHspB1WT system, the ratio of minor components was up to 18%, which depended on both temperature and concentration. Referring to the previous study [26], *f*/*f*_0_ could have an error of 5–10% in the CgHspB1WT system. Assuming that *f*/*f*_0_ could have the error of 5–10%, the error in the molecular weight and association number was calculated to be 7.5–15% based on Equation (1).

At 4 °C, CgHspB1 wild-type (CgHspB1WT) at the 1.5 mg/mL concentration appeared as a single peak with a sedimentation coefficient of 13.6 S (Figure 1A). The molecular mass was estimated to be 447 kDa, which corresponds to a 19-mer. Then, we examined the effect of the concentration change. The sedimentation coefficient was only partially affected by the dilution. The peak appeared almost at the same positions, 13.5 S (395 kDa, 17-mer) at 0.5 mg/mL and 13.9 S (447 kDa, 19-mer) at 0.1 mg/mL; therefore, we concluded that CgHspB1 WT exists as an 18-mer at 4 °C. Peak broadening was observed in diluted conditions. Interestingly, small and larger oligomers appeared in diluted conditions, suggesting that the oligomeric structures are in dynamic equilibrium with other oligomeric structures. At 25 °C, the sedimentation coefficient slightly increased at all concentrations (Figure 1B). The sedimentation coefficient was calculated to be 15.0 S (460 kDa, 20-mer) at 1.5 mg/mL. The main peaks appeared at almost the same positions at the diluted conditions, 15.1 S at 0.5 mg/mL and 15.0 S at 0.1 mg/mL. Further, the friction ratio did not show a meaningful change. It is difficult to tell whether it was caused by the change of molecular mass or the shape. At 4 °C, the peak was broadened and additional peaks appeared at the lower concentration. At 40 °C, a significant change was observed (Figure 1C); the sedimentation coefficient had increased to 18.4 S (623 kDa, 27-mer). Under the diluted condition, several peaks for the dissociated small oligomers appeared. Small oligomers seem to be dimers, hexamers, dodecamers, and octadecamers.

**Figure 2:**
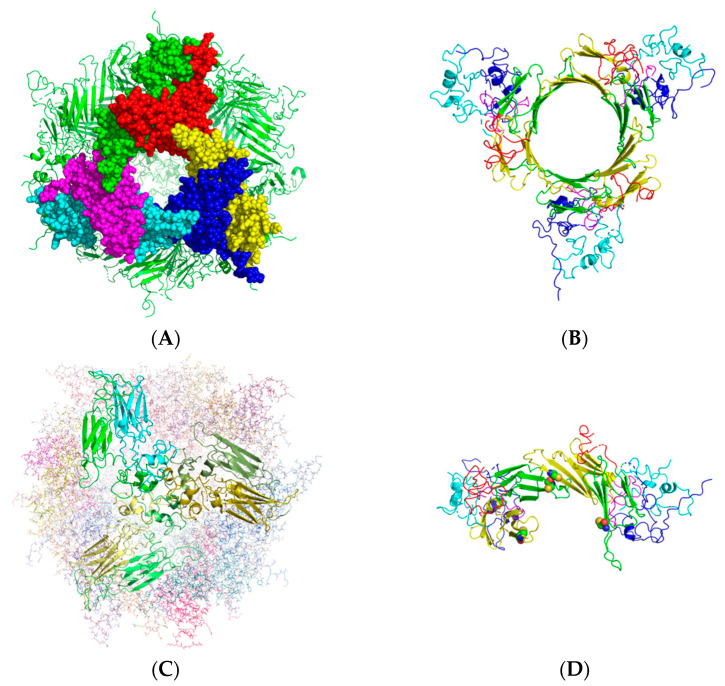
Crystal structure of human HspB1. (**A**) 24-mer structure. A hexamer is shown as the space-filling model. (**B**) Structure of a hexamer. Blue and Cyan—NTDs, Green and Yellow—α-crystallin domains, Red and Magenta—CTEs. (**C**) Three hexamers were assembled by the interaction of NTDs. Dimers in three hexamers, shown as ribbon models, interact via NTDs (ribbon model). (**D**) Location of cysteine residues at the interface between dimers. The structures of the two dimers are shown in the ribbon model. Cysteine residues are shown as space-filling model.

Recently, the crystal structure of human HspB1 was revealed to be a 24-mer composed of 4 hexamers (Figure 2A) [23]. The complex is composed of 4 hexamers. Three beta strands of each subunit in the hexamer constitute the 18-stranded beta-barrel structure. The hexamer consists of three dimers (Figure 2B). There is a 2-fold relationship between identical monomers within the dimer, leading to very compact structural packing. The N-terminal domain seems to be responsible for the assembly of the dimer. The N-terminal domain is also involved in the assembly of hexamers (Figure 2C). The N-terminal domain complex of each dimer unit assembles with those of two dimers in other hexametric units. The conserved cysteine residues reside at the dimer interface (Figure 2D), suggesting a role in hexamer formation. Among the three phosphorylated serine residues under stress conditions [22], only S15 is in the hexameric assembly of N-terminal domains. Two S15 residues face each other, suggesting that phosphorylation of S15 affects the stability of hexamer assembly. S78 and S82 are positioned in a common surface cavity.

It is plausible that CgHspB1 exists as 24-mer at 40 °C. It is in dynamic equilibrium with the structures in the hexameric units. The hexamer also dissociates to dimers. The temperature-dependent change was reversible (Supplementary Figure S1 and Table S2). By the temperature shift from 40 °C to 4 °C, the sedimentation coefficient of the oligomer decreased close to that value at 4 °C.

**Figure 3:**
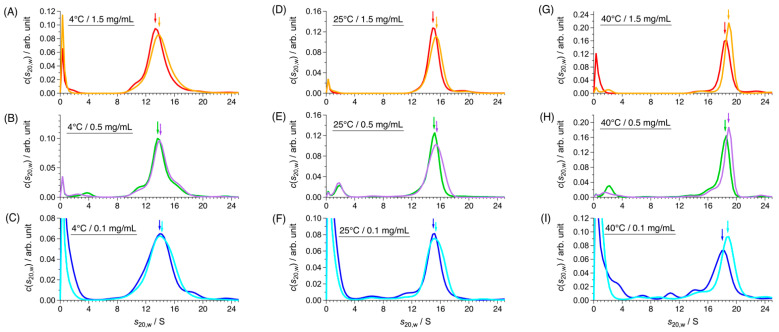
SV-AUC results of CgHspB1 WT and CgHspB1 S15D at (**A**–**C**) 4 °C, (**D**–**F**) 25 °C, and (**G**–**I**) 40 °C. Red, green, and blue lines represent *c*(*s*_20,w_) of CgHspB1 WT at 1.5 mg/mL, 0.5 mg/mL, and 0.1 mg/mL, respectively. Yellow, purple, and cyan lines represent *c*(*s*_20,w_) of CgHspB1 S15D at 1.5 mg/mL, 0.5 mg/mL, and 0.1 mg/mL, respectively. Colored arrows corresponding to the data with the same colors show the main peak positions in *c*(*s*_20,w_) and their parameters are listed in Table 2.

Previously, we showed that a phosphorylation mimic mutant of CgHspB1 with the replacement of Ser15 with Asp (CgHspB1S15D) exhibited relatively lower oligomer stability and more remarkable protective ability against thermal aggregation of citrate synthase from the porcine heart (CS) than the wild type [17]. In SV-AUC, the CgHspB1S15D mutant showed almost identical results as CgHspB1WT (Figure 3). Although we expected to observe more dissociated conformers in CgHspB1S15D at 40 °C, there was virtually no difference. Thus, it is unreasonable to think that the dissociated conformation is the active conformation. The sedimentation coefficient of CgHspB1S15D was slightly larger than that of CgHspB1WT, suggesting partial conformational change.

### 2.2. Role of the Disulfide Bond

Chalova et al. showed that oxidative stress, inducing the formation of disulfide bonds, can affect the stability and conformational mobility of human HspB1 [27]. A previous report showed that Hsp25, murine HspB1, can exist within the cell as a covalently bound dimer linked by an intermolecular disulfide bond between two monomers [28]. The formation of the intermolecular disulfide bond did not affect the overall secondary structure, the degree of oligomerization, or the chaperone activity of Hsp25. Curiously, in the crystal structure of human HspB1, the conserved Cys residue is not involved in dimeric unit formation but seems to be responsible for the assembly of dimers to a hexamer (Figure 2D).

**Figure 4:**
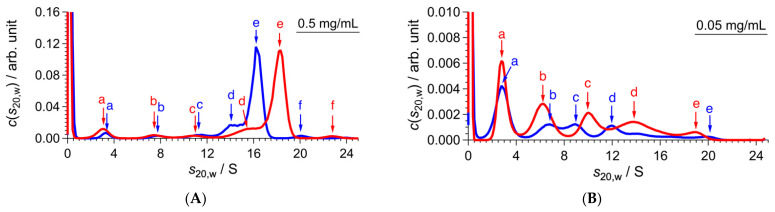
SV-AUC results of CgHspB1 WT with and without DTT at 40 °C. Blue and red lines represent *c*(*s*_20,w_) with and without DTT, respectively. (**A**,**B**) show the results at 0.5 mg/mL and 0.05 mg/mL, respectively. Colored arrows and letters (a, b, c, d, e, f) corresponding to the data with the same colors show the peak positions whose parameters are listed in Table 3.

To elucidate the role of the disulfide bond, we examined the effect of dithiothreitol (DTT) on the oligomer conformation of CgHspB1WT at 40 °C (Figure 4). At a concentration of 0.5 mg/mL, the addition of DTT significantly affected the oligomeric structure. The sedimentation coefficient decreased from 18.3 S to 16.2 S. The 24-mer structure might be affected by the loss of disulfide bonds. At 0.05 mg/mL, the large oligomer of CgHspB1WT almost wholly dissociated into small oligomers. Without DTT, they seem to be dimers, hexamers, dodecamers, and octadecamers. In the presence of DTT, the proportion of dimers increased, suggesting that disulfide bonds between dimers partially stabilize hexamers. Therefore, the difference in oligomeric structure should be caused by the loss of the disulfide bond.

**Figure 5:**
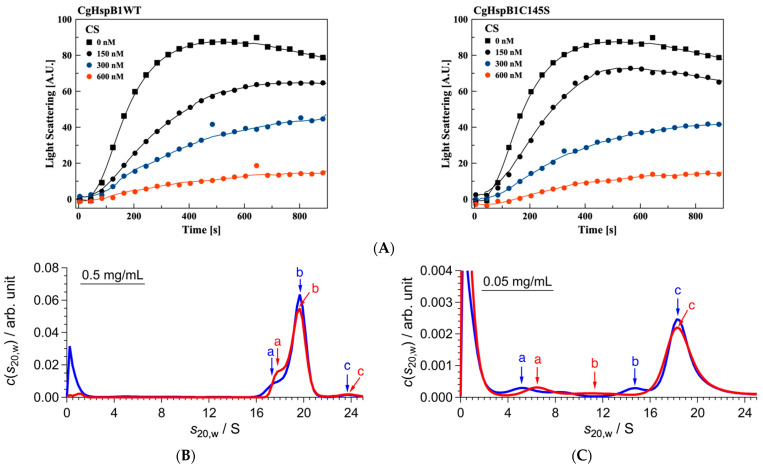
Structure and function of CgHspB1 C145S. (**A**) Comparison of the effects of CgHspB1C145S and CgHspB1WT on the thermal aggregation of citrate synthase. The thermal aggregation of CS from the porcine heart was monitored by measuring light scattering at 500 nm with a spectrofluorometer at 50 °C. CS (50 nM, monomer) was incubated in assay buffer with or without CgHspB1WT and CgHspB1 C145S (150, 300, and 600 nM as monomers). (**B**,**C**) SV-AUC results of CgHspB1 C145S with and without DTT at 40 °C. Blue and red lines represent *c*(*s*_20,w_) with and without DTT, respectively. (**B**,**C**) show the results at 0.5 mg/mL and 0.05 mg/mL, respectively. Colored arrows and letters (a, b, c) corresponding to the data with the same colors show the peak positions whose parameters are listed in Table 4.

Then, we made a mutant with the amino acid replacement of Cys145 to Ser (CgHspB1C145S). CgHspB1C145S exhibited almost the same chaperone activity on heat-denatured citrate synthase (Figure 5A). The oligomeric structure was analyzed by SV-AUC (Figure 5B). At 40 °C, it appeared at 19.7 S in AUC, slightly larger than the wild type. The change in the sedimentation coefficient should reflect conformational changes because *f*/*f*_0_ decreases without changing the molecular weight. The CgHspB1C145S oligomer seems to be slightly compact compared with the CgHspB1WT oligomer. Corresponding to the loss of the cysteine residue, the sedimentation coefficient of the oligomer was not affected by the addition of DTT. Contrary to the observation in CgHspB1WT, dissociation of the oligomer was not observed at a concentration of 0.05 mg/mL (Figure 5C). However, the sedimentation coefficient slightly decreased to show conformational change. Thus, the mutation of C145S affected the conformation not only by the loss of the disulfide bond. We also attempted to express the C145G mutant. However, it could not fold correctly in *E. coli* and was obtained as an inclusion body. Since Cys 145 is located at the interface between dimers in the hexamer, mutation of Cys 145 should affect oligomer assembly or stability.

### 2.3. Effects of N- and C-Terminus Truncations

**Figure 6:**
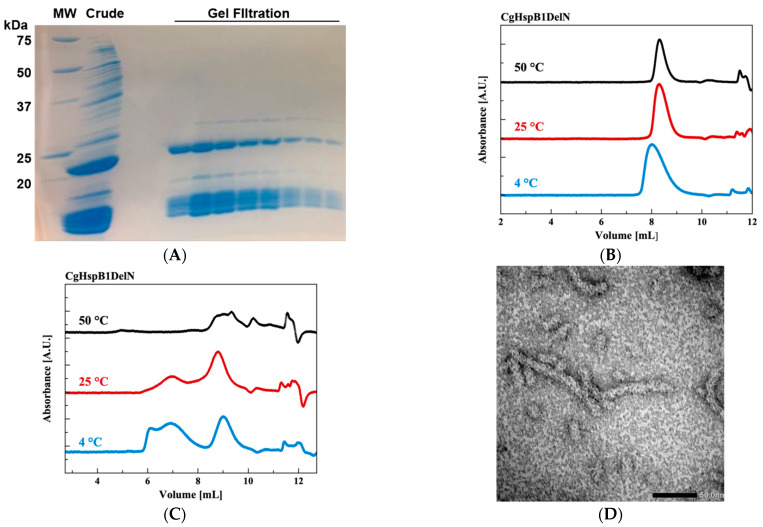
Characterization of CgHspB1DelN and CgHspB1DelC. (**A**) SDS–PAGE of CgHspB1DelN. MW—molecular weight marker, Crude—collected fractions of 1-st anion exchange chromatography, Gel-filtration—fractions of gel filtration chromatography. (**B**) HPLC-SEC analysis of CgHspB1DelN. (**C**) HPLC-SEC analysis of CgHspB1DelC. (**D**) Electron microscopy image of CgHspB1DelC.

We tried to reveal the roles of NTD and CTE. First, we made a CgHspB1 N-terminal deletion mutant (CgH-spB1DeltaN) in which the N-terminal 30 amino acids were deleted. It was expressed in *E. coli* using the pET23b vector. CgHspB1DeltaN was susceptible to protease digestion in *E. coli* and appeared as digested bands in SDS–PAGE (Figure 6A). However, curiously, CgHspB1DeltaN was obtained as a uniform oligomer, probably a hexamer (Figure 6B). Assuming that CgHspB1DeltaN was digested by the protease in *E. coli*, we attempted to express it at the lowered temperature using the pCold vector. In this condition, it was expressed without digestion. However, it was produced as a large complex. It was not resolved by the addition of 20% ethylene glycol (data not shown). We thought the remaining N-terminal region would induce aggregation of CgHspB1DeltaN. Then, we attempted to remove the N-terminal region, but it was not expressed in *E. coli* (data not shown). The location of the CTE is interesting in the crystal structure. One CTE of a dimer is involved in hexamer assembly, and the other CTE is not. The purified CTE deletion mutant appeared at various positions in SEC (Figure 6C). They appeared as filamentous structures in EM (Figure 6D). Thus, the C-terminal deletion mutant was unable to assemble hexamers. The exposed hydrophobic surface might cause the formation of large oligomers.

## 3. Discussion

The oligomeric structure of HspB1 changes with temperature and concentration. Previously, we reported that CgHspB1 assembles into a 16-mer and dissociates into dimers at elevated temperatures. However, the present SV-AUC results contradict previous observations. CgHspB1 oligomer was found to be 18-mer at 4 °C by SV-AUC. The oligomer partially dissociates under diluted conditions. As the temperature increases, the sedimentation coefficient increases. At 40 °C, CgHspB1 seems to exist as a 24-mer, which should correspond to the crystal structure of human HspB1. The peaks are broadened at the diluted conditions. At 0.05 mg/mL concentration, the large oligomer almost diminished, and small oligomers appeared. They seem to be dimers, hexamers, 12-mers, and 18-mers. The results clearly show that HspB1 exists as various oligomeric structures. We speculate that our previous observation of 16-mer should be due to the coexistence of multiple oligomers.

**Figure 7:**
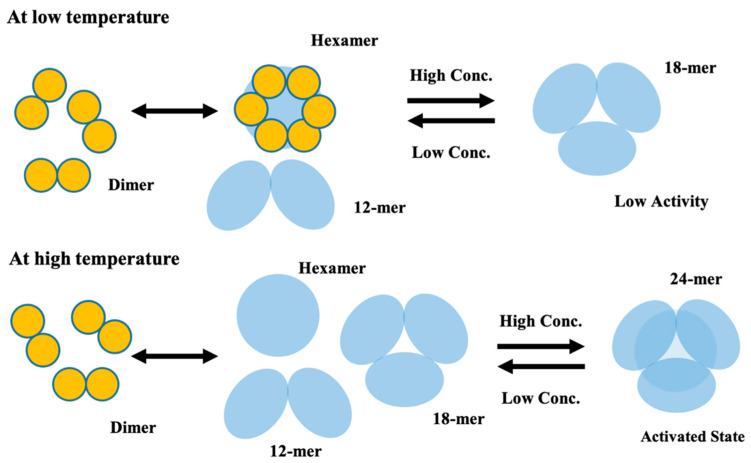
Schematic conformation transition model of CgHspB1.

Figure 7 shows a schematic model of the conformational change of CgHspB1. At the low temperature of 4 °C, CgHspB1 exists as an 18-mer, probably a trimeric complex of hexamers. It is relatively unstable and partially dissociates into small oligomers, hexamers, and dodecamers. In addition, hexamers may assemble not only into 18-mers but also 24-mers. However, at lower temperatures, the 24-mer is relatively unstable compared with the 18-mer. At elevated temperatures, the 24-mer was more stable than the 18-mer. The 24-mer is also in dynamic equilibrium with the dissociated oligomers in the hexameric unit. In addition, the hexamer dissociates to dimers. It is difficult to determine how the sedimentation coefficient increased at 25 °C. As the *f/f_0_* value was slightly decreased at 25 °C, the increase in the sedimentation coefficient might be caused by the conformational change. Another possibility is the coexistence of 18-mer and 24-mer in equilibrium.

Previously, we speculated that the dimer is the structural and functional unit of sHsp. The crystal structures of sHsps or archaea, fission yeast, and wheat show that dimers assemble into large oligomers by the interaction between the C-terminal IXI/V motif and the α-crystallin domain. At elevated temperatures, they disassemble to dimers. The crystal structure of human HspB1 and our results do not fit the model. The structure unit is a hexamer, and the IXI motif does not seem to be responsible for oligomer assembly. It is generally thought that sHsps express chaperone functions in the dissociated dimeric conformation. However, there have been various contradicting results. An Hsp26 variant with a cysteine residue at the N-terminus does not dissociate into dimers, but the chaperone activity remains unaffected under oxidized conditions [29]. Previously, we made various mutants of StHsp14.0 and sHsp of *Sulfolobus tokodaii* [30,31,32]. Among them, the IXI/V motif mutants StHsp14.0WKW and StHsp14.0FKF dissociated into dimers at relatively low temperatures compared with the wild type. At 50 °C, StHsp14.0FKF dissociated to dimers completely, while StHsp14.0WKW dissociated partially. However, the chaperone activity of StHsp14.0FKF was weaker than that of StHsp14.0WKW. Moreover, the CgHspB1 oligomer does not dissociate into small oligomers at relatively high concentrations [17]. In this study, we showed that CgHspB1C145S did not dissociate at 40 °C, even under diluted conditions. Based on these facts, we concluded that the active conformer of CgHspB1 is not dissociated small oligomers, dimers, or hexamers. It is most plausible that the 24-mers observed at 40 °C are the active conformation of CgHspB1. For the moment, we have no clear idea how 24-meric CgHspB1 interacts with unfolded proteins to protect them from aggregation formation. Since the spherical 24-mer has a large central cavity with accessible holes, it is plausible that the unfolded peptide is captured in the cavity. It is also unknown why 18-mer does not have a chaperone function because its structure is unsolved. The central cavity or hole may be smaller compared with 24-mer.

## 4. Materials and Methods

### 4.1. Cloning, Expression, and Purification

Wild-type CgHspB1 and the phosphorylated mimics CgHspB1 and CgHspB1S15D were expressed in *E. coli* BL21 Star (DE3) and purified as described previously [17]. The plasmids used to produce CgHspB1C145S and CgH-spB1C145G were made through site-directed mutagenesis with the primers shown in Supplementary Table S1 using pET23b-CgHspB1WT as a template. The N-terminal and C-terminal deletion mutant genes were made by PCR using the primers shown in Table 1 and then cloned in pET23b by NdeI and XhoI. *E. coli* BL21 (DE3) cells transformed with plasmids to produce CgHspB1 variants were grown at 37 °C in Luria–Bertani medium containing 100 µg/mL ampicillin for 24 h. The cells were harvested by centrifugation at 5000× *g* for 10 min at 4 °C. The harvested cells were suspended in buffer A (50 mM Tris–HCl, pH 8.0) and disrupted by sonication, and the suspension of disrupted cells was centrifuged at 24,000× *g* for 30 min at 4 °C. The supernatant was applied to a TOYOPEARL DEAE-650 anion exchange column (Tosoh, Tokyo, Japan) equilibrated with buffer A. Proteins were eluted with a linear gradient of 0–400 mM NaCl in buffer A. Fractions containing CgHspB1 were pooled and dialyzed with buffer A overnight. The dialyzed protein solution was applied to a RESOURCE Q column (GE Healthcare Bio-Sciences, Buckinghamshire, England) equilibrated with buffer A. Proteins were eluted with a linear gradient of 0–500 mM NaCl in buffer A. Fractions containing CgHspB1 were pooled, concentrated by ultrafiltration (Amicon Ultra, Merck Millipore, Billerica, CA, USA), and then applied to a HiLoad 26/60 Superdex 200-pg size-exclusion column (GE Healthcare Bio-Sciences, Buckinghamshire, England) equilibrated with buffer B (50 mM Tris-HCl pH 7.5, 0.1 mM EDTA, 150 mM NaCl).

### 4.2. Protein Aggregation Measurements

The thermal aggregation of citrate synthase from the porcine heart (CS) was monitored by measuring light scattering at 500 nm with a spectrofluorometer (FP-6500, JASCO, Tokyo, Japan) at 50 °C as described previously [31]. Native CS (50 nM, monomer) was incubated in TKM buffer (50 mM Tris-HCl, pH 7.5, 100 mM KCl, and 25 mM MgCl_2_) with or without CgHspB1WT or CgHspB1C145S. The assay buffer was preincubated at 50 °C and continuously stirred throughout the measurement.

### 4.3. HPLC-SEC

HPLC-SEC was performed with a gel-filtration column (SB-804HQ, Showa Denko, Tokyo, Japan) using an HPLC system, PU-1580i, connected to an MD1515 multiwavelength detector (JASCO), as described previously [17]. CgH-spB1WTDelN or CgHspB1WTDelC was heated at the specified temperature for 30 min, loaded onto a column heated at the same temperature, and eluted with buffer B with or without 20% ethylene glycol at a flow rate of 1.0 mL/min. The proteins were monitored by the absorbance at 215 nm.

### 4.4. SV-AUC Measurements

SV-AUC measurements were carried out with ProteomeLab XL-I (Beckman Coulter, Brea, CA, USA). Samples were filled in 12 mm-pathlength Epon double sector centerpieces. All measurements were performed using Rayleigh interference optics at a 40,000-rpm rotor speed. The time evolution of sedimentation data was analyzed with the Lamm formula. Then, the weight concentration distribution of components, *c*(*s*_20,w_), was obtained as a function of the sedimentation coefficient. Here, the sedimentation coefficient was normalized to be the value at 20 °C in pure water, *s*_20,w_. The molecular weight *M* and association number *N* of each component were calculated using the following equation:

(1) $$M={\left[\frac{6\pi \eta N_{A}}{\left(1-\rho \bar{v}\right)}\right]}^{1.5}{\left(\frac{3\bar{v}}{4\pi N_{A}}\right)}^{0.5}{\left(\frac{f}{f_{0}}\right)}^{1.5}s_{20,w}{}^{1.5}\;,\;\;\;\;\;$$

(2) $$\;\;N=\frac{M}{M_{1}}\;,$$

where ρ, η, $\bar{v}$,$\;N_{A}$, $f/f_{0}$, and $M_{1}$ are the solvent density, solvent viscosity, partial specific volume, Avogadro number, friction ratio, and molecular weight of a monomer, respectively. These calculations were performed with SEDFIT software [33]. The density and viscosity of solvents were measured with a density meter DMA4500M (Anton Paar, Graz, Austria) and a viscometer Lovis 2000 M/ME (Anton Paar, Graz, Austria), respectively.

### 4.5. Electron Micrograph 

Samples were applied to carbon-coated copper grids and negatively stained with 2.5% (*w*/*v*) uranyl acetate. Micrographs were recorded at a magnification of 50,000× with a JEM-1400 transmission electron microscope (JEOL, Tokyo, Japan) operated at 120 kV. 

## Figures and Tables

**Table 1:** Parameters of peak positions of *c*(*s*_20,w_) in Figure 1.

**(A) 4 °C**					
***c*/mg mL^−1^**	***f/f*_0_**	**Peak**	***s*_20,w_/S**	***M*/kDa**	**Association Number**
1.5	1.40	a	10.5	266	11
		**b**	**13.5**	**395**	**17**
		c	19.8	688	30
0.5	1.46	a	3.7	55	2
		b	11.1	310	13
		**c**	**13.6**	**447**	**19**
		d	19.8	552	24
0.1	1.44	a	7.8	176	7
		**b**	**13.9**	**447**	**19**
		c	18.5	653	28
**(B) 25 °C**					
***c*/mg mL^−1^**	***f/f*_0_**	**Peak**	***s*_20,w_/S**	***M*/kDa**	**Association Number**
1.5	1.43	**a**	**15.0**	**460**	**20**
		b	19.6	694	30
0.5	1.39	a	1.8	20	1
		b	6.3	131	6
		**c**	**15.1**	**432**	**18**
0.1	1.41	a	6.3	133	6
		b	11.5	308	13
		**c**	**15.0**	**447**	**19**
**(C) 40 °C**					
***c*/mg mL^−1^**	***f/f*_0_**	**Peak**	***s*_20,w_/S**	***M*/kDa**	**Association Number**
1.5	1.42	a	15.1	468	20
		**b**	**18.4**	**623**	**27**
		c	22.6	885	38
0.5	1.36	a	2.2	24	1
		b	7.6	151	6
		c	13.6	334	14
		d	16.4	491	21
		**e**	**18.5**	**567**	**24**
		f	23.0	832	36
0.1	1.40	a	3.2	43	2
		b	6.9	143	6
		c	10.7	227	12
		d	14.2	405	17
		**e**	**18.2**	**598**	**26**
		f	21.9	790	34

Bold characters indicate main component in *c*(*s*_20,w_).

**Table 2:** Parameters of main peak of *c*(*s*_20,w_) in Figure 3.

**(A) 4 °C**					
**Sample**	***c*/mg mL^−1^**	***f/f*_0_**	***s*_20,w_/S**	***M*/kDa**	**Association Number**
CgHspB1 WT	1.5	1.40	13.5	395	17
CgHspB1 S15D		1.45	13.9	427	18
CgHspB1 WT	0.5	1.46	13.6	447	19
CgHspB1 S15D		1.44	13.9	449	19
CgHspB1 WT	0.1	1.44	13.9	447	19
CgHspB1 S15D		1.43	14.0	435	19
**(B) 25 °C**					
**Sample**	***c*/mg mL^−1^**	***f/f*_0_**	***s*_20,w_/S**	***M*/kDa**	**Association Number**
CgHspB1 WT	1.5	1.43	15.0	460	20
CgHspB1 S15D		1.35	15.4	443	19
CgHspB1 WT	0.5	1.39	15.1	432	18
CgHspB1 S15D		1.39	15.4	435	19
CgHspB1 WT	0.1	1.41	15.0	447	19
CgHspB1 S15D		1.34	15.1	413	18
**(C) 40 °C**					
**Sample**	***c*/mg mL^−1^**	***f/f*_0_**	***s*_20,w_/S**	***M*/kDa**	**Association Number**
CgHspB1 WT	1.5	1.42	18.4	623	27
CgHspB1 S15D		1.40	18.9	628	27
CgHspB1 WT	0.5	1.36	18.5	567	24
CgHspB1 S15D		1.39	18.9	609	26
CgHspB1 WT	0.1	1.40	18.2	598	26
CgHspB1 S15D		1.35	18.9	597	26

**Table 3:** Parameters of peak positions of *c*(*s*_20,w_) in Figure 4.

**0.5 mg/mL**					
**DTT**	***f/f*_0_**	**Peak**	***s*_20,w_/S**	***M*/kDa**	**Association Number**
+	1.42	a	3.3	47	2
		b	7.7	169	7
		c	11.2	299	13
		d	14.0	420	18
		**e**	**16.2**	**518**	**22**
		f	20.0	713	30
−	1.40	a	3.1	43	2
		b	7.5	161	7
		c	10.9	282	12
		d	15.5	475	20
		**e**	**18.3**	**612**	**26**
		f	22.7	845	36
**0.50 mg/mL**					
**DTT**	***f/f*_0_**	**Peak**	***s*_20,w_/S**	***M*/kDa**	**Association Number**
+	1.45	a	2.8	38	2
		b	6.6	139	6
		c	8.9	217	9
		d	11.9	336	14
		e	20.1	738	32
−	1.47	a	2.8	39	2
		b	6.3	132	6
		c	10.0	264	11
		d	13.8	428	18
		e	18.9	687	29

Bold characters indicate main component in *c*(*s*_20,w_). “+” and “-” mean the addition and non-addition of DTT, respectively.

**Table 4:** Parameters of peak positions of *c*(*s*_20,w_) in Figure 5B.

**0.5 mg/mL**					
**DTT**	***f/f*_0_**	**Peak**	***s*_20,w_/S**	***M*/kDa**	**Association Number**
+	1.25	a	17.5	484	21
		**b**	**19.7**	**575**	**25**
		c	23.7	760	32
−	1.26	a	17.8	495	21
		**b**	**19.7**	**576**	**25**
		c	23.7	762	33
**0.50 mg/mL**					
**DTT**	***f/f*_0_**	**Peak**	***s*_20,w_/S**	***M*/kDa**	**Association Number**
+	1.26	a	5.2	79	3
		b	14.6	375	16
		**c**	**18.2**	**523**	**22**
−	1.27	a	6.4	109	5
		**b**	**18.2**	**523**	**22**

Bold characters indicate main component in *c*(*s*_20,w_). “+” and “-” mean the addition and non-addition of DTT, respectively.

## Supplementary Materials

The following are available online at https://www.mdpi.com/article/10.3390/ijms221910797/s1.

## Abbreviations

ACD	α-crystallin domain
NTD	N-terminal domain
CTE	C-terminal extension
SEC	Size-exclusion chromatography
SEC-MALS	Size-exclusion chromatography with multiangle light scattering
SAXS	Small-angle X-ray scattering
SV-AUC	Sedimentation velocity analytical ultracentrifugation
CgHspB1	HspB1 from Chinese hamster
CgHspB1WT	CgHspB1 wild type
CgHspB1C145S	CgHspB1 C145S mutant
CgHspB1DelN	CgHspB1 NTD deletion mutant
CgHspB1DelC	CgHspB1 CTE deletion mutant
DTT	Dithiothreitol
CS	Citrate synthase from porcine heart
