# Supplementary material for: Oligomeric Structural Transition of HspB1 from Chinese Hamster
Source: Int J Mol Sci. 2021 Oct 6;22(19):10797. doi: 10.3390/ijms221910797 (PMC8509488; doi:10.3390/ijms221910797)
Supplement: Supplementary file 1 [file ijms-22-10797-s001.zip › ijms-1398551-supplementary.pdf]

Supplementary Table S1 Primes used to construct CgHspB1 variants

Product	Fw/Rv	Sequence
CgHspB1C145S	Fw	5’- TCCCGGAGTTTTACCCGGAAATACACG-3’
	Rv	5’- GGTAAAGCTCCGGGAGATGTAGCCATG-3’
CgHspB1_delN	Fw	5’-CCCATATGGACCAAGCCTTCGGG-3’
	Rv	5’- CCCTCGAGCTACTTGGCTCCAGACTGTTC-3’
CgHspB1_delC	Fw	5’- CGCCATATGACCGAGCGCCGC-3’
	Rv	5’- CCCTCGAGCTAGACCGGAAT GGTGATCTC-3’

Supplementary Fig. S1

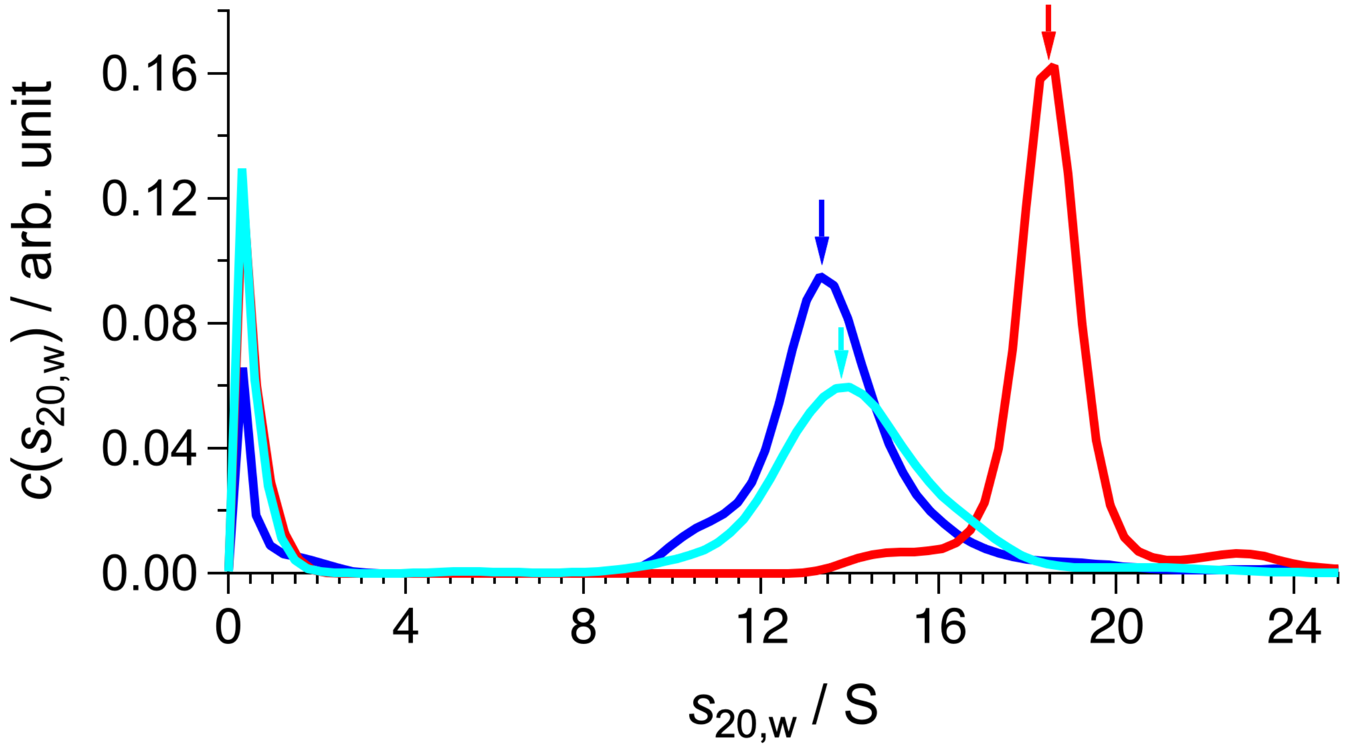

SV-AUC results of CgHspB1 WT depending on the temperature history. Blue and red lines represent $c(s_{20,w})$ at 4 °C and 40 °C, respectively. The cyan line shows $c(s_{20,w})$ at 4 °C after incubation at 40 °C for 2 hours. Colored arrows corresponding to the data with the same colors indicate the peak positions whose parameters are listed in Table S2.

Supplementary Table S2. Parameters of peak positions of $c(s_{20,w})$ in Figure S1.

Temperature/°C	c/mg mL^{-1}	f/f_0	$s_{20,w}$/S	M/kDa	Association number
4	1.5	1.40	13.5	395	17
40	1.5	1.42	18.4	623	27
4 (after incubation at 40 °C)	1.5	1.41	14.3	433	19
